# Publicity and Ethics

**Published:** 1915-11

**Authors:** 


### Publicity and Ethics.

Within the last few years, the issue has arisen with regard to advertising by physicians, in a very definite form. Does the social standing, professional prominence or prestige of a medical man exempt him from the ordinary rules of medical ethics? This problem has even been presented in a still more definite form, namely, as to the standing of a man making obvious effort to secure publicity, not by direct paid newspaper advertising and the like, but by precisely the same indirect means as are employed by the elect, the elect not considering him a proper person for acquiring prestige. That many prominent men do maintain a publicity bureau, quite comparable except as to details with those of business firms, expositions, etc., is well known. In some cases, the bureau deals with intra-professional publicity, in others a pretty direct appeal to the laity is made though, in the latter case, the circumstances are often, perhaps usually such as to preclude the idea of financial gain. On the contrary, mere glory seems to be the objective. Occasionally it has happened that publicity, either striven for or forced upon the individual has reacted to his detriment, so that he has been forced to take steps to prevent such publicity. Recently, we have received a marked copy of a lay magazine containing an illustrated write-up of a prominent member of the profession, outside our territory. It is just such a write-up as might be expected to cater to public curiosity regarding the private life of kings, presidents, etc. We do not see how such publicity can help the individual; rather it tends to diminish the esteem in which he has properly been held by the profession and to lessen calls upon him by his colleagues. It can only be desired by the individual as a gratification of personal vanity at the cost of professional reputation and income.

Some months ago, a county medical society in a distant part of the country, protested vigorously against the assumption of one of its most renowned members that his position exempted him from the ordinary rules of ethics in regard to publicity. This was a courageous and sensible action and should lead to general discussion of the subject and the establishment of fairly definite principles, among them the provision that ethics is the same thing for all members of the profession. The great evil of a medical aristocracy, exempt from ethical rules, is the temptation placed in the way of young and inexperienced members of the profession to whom publicity appears not merely as a gratification of vanity but as a means of increasing their earnings.

